# Impact of COVID-19 pandemic: increase in complicated upper respiratory tract infections requiring ENT surgery?

**DOI:** 10.1007/s00405-023-08349-3

**Published:** 2023-12-12

**Authors:** Jonas Galli, Sean C. Sheppard, Marco Caversaccio, Lukas Anschuetz, Sven Beckmann

**Affiliations:** Department of Otorhinolaryngology, Head and Neck Surgery, Inselspital, Bern University Hospital, University of Bern, Freiburgstrasse 18, 3010 Bern, Switzerland

**Keywords:** COVID-19 pandemic, Upper respiratory tract infections, Surgical intervention, Upper respiratory tract infection-related complications, Microbial profile

## Abstract

**Purpose:**

This study investigates the impact of the COVID-19 pandemic on complicated upper respiratory tract infections requiring surgical intervention in a tertiary referral center. The aim is to understand the consequences of pandemic-related measures and their subsequent relaxation on the incidence and characteristics of upper respiratory tract infection-related complications.

**Methods:**

Patients who underwent surgery as a complication of upper respiratory tract infections between December 2014 to February 2023 were included. Demographic information, surgical procedures, microbiological findings, and clinical outcomes were assessed and analyzed comparing pre-pandemic, pandemic and post-pandemic groups.

**Results:**

321 patients were enrolled, including 105 patients (32.7%) in the pediatric population. Comparison of pre-pandemic (*n* = 210), pandemic (*n* = 46) and post-pandemic periods (*n* = 65) revealed a statistically significant increase in complicated otologic infections requiring surgical intervention in the post-pandemic period compared to the pandemic period (*p* value = 0.03). No statistically significant differences in other surgical procedures or demographic parameters were observed. A statistically significant increase in urgent ear surgery in the pediatric population between the pandemic and the post-pandemic period (*p* value = 0.02) was observed. Beta-hemolytic group A streptococcal infections showed a statistically significant increase in the post-pandemic period compared with the pandemic period (*p* value = 0.02).

**Conclusions:**

Relaxation of COVID-19-related restrictions was associated with an increase of upper respiratory tract infection-related otologic infections requiring surgical intervention with an increasing rate of beta-hemolytic group A streptococcal infections. These findings highlight the importance of considering the impact of the pandemic on upper respiratory tract infection complications and adapting management strategies accordingly.

## Introduction

With the worldwide spread of the coronavirus disease 2019 (COVID-19) due to infections with SARS-CoV-2, numerous pandemic-related measures were imposed. SARS-CoV-2 belongs to the family of enveloped single-stranded RNA viruses known as coronaviruses and is primarily transmitted through respiratory droplets and aerosols with active virus replication in the upper respiratory tract [1, 2]. From February 2020 onwards, when the pandemic reached Europe, the Swiss government implemented various restrictions, including mandatory masks, social distancing measures and lockdowns to slowdown the spread of COVID-19. Consequently, restrictions during this period led to an initial decline in otorhinolaryngological consultations [3]. Furthermore, the occurrence of upper respiratory tract infections such as acute laryngitis, acute tracheitis and acute otitis media was significantly decreased [4–6]. As the pandemic continued to progress and the number of new infections was gradually reduced, these restrictions were lifted by the Swiss Federal Council on April 1, 2022 [7]. Typically, the winter months of December to February are associated with an increase in upper respiratory tract infection cases in the Northern Hemisphere, due to cold temperatures and low humidity [8]. While viral infections of the upper respiratory tract are common, subsequent bacterial infections are relatively rare [9, 10]. However, if not properly managed, these infections can lead to serious complications and long-lasting sequelae [10].

We observed a post-pandemic increased incidence of complicated upper respiratory tract infections requiring surgical intervention, which prompted us to investigate the rate in our tertiary referral center before, during and after the COVID-19 pandemic. In addition, we aimed to assess if there was a general increase in infections or a shift in the bacterial spectrum. By studying the impact of the COVID-19 pandemic on complicated upper respiratory tract infection rates and the microbiological profile of infections, we hope to gain valuable insights into the consequences of pandemic-related measures and their subsequent relaxation. Understanding these trends is essential to inform healthcare practice, improve patient management and address potential challenges in the prevention and treatment of complicated upper respiratory tract infections.

## Materials and methods

### Patients

We retrospectively analyzed the surgical records of a tertiary referral center in Switzerland (Inselspital, Bern University Hospital) for the 3 months of December, January and February from December 2014 to February 2023. All patients requiring surgical intervention in the operating room (OR) by the ENT department as a complication of an upper respiratory tract infection were included in this study, regardless of age and type of anesthesia (local or general). Patients with necrotizing fasciitis, superinfected neck cysts or superinfected head and neck cancer were excluded. Patient age, imaging modalities, surgical procedures, microbiological findings and outcome were recorded and analyzed in a database. For statistical analysis, patients were divided into pre-pandemic (December 2014 to February 2020), pandemic (December 2020 to February 2022) and post-pandemic (December 2022 to February 2023) groups.

Statistical analysis was performed with a Kruskal–Wallis test between the pre-pandemic, pandemic and post-pandemic groups and plotted with GraphPad Prism 9 (GraphPad Software, San Diego, California, USA). In case of a statistically significant difference, Dunn's test was performed to determine which groups showed statistically significant differences. Statistically significant difference was assumed for a two-tailed alpha-value of < 0.05.

## Results

A total of 321 patients were enrolled in this study, including 105 pediatric patients (32.7%) with < 18 years of age. The mean age was 32.8 years (± 23.0 years) with 129 females (40.2%). Detailed demographic features of the pre-pandemic, pandemic and post-pandemic groups are summarized in Table 1. Overall, 59.5% of patients underwent computed tomography, 16.2% of patients received MR tomography, and 14.6% of patients required both computed and MR tomography.

**Table 1:** Demographic data of the included subjects

	Pre-pandemic (2014–2020)	Pandemic (2021–2022)	Post-pandemic (2023)
Number of patients	210	46	65
Mean age and SD (years)	35.0 ± 22.3	34.8 ± 24.6	24.2 ± 22.7
Male	125	27	40
Female	85	19	25

*SD* standard deviation

After separation into pediatric and adult population, there was an increased rate of MR tomography (40% vs 4.6%) and a reduced rate of computed tomography (36.2% vs 71.3%) in pediatric population compared to the adult population.

**Fig. 1:**
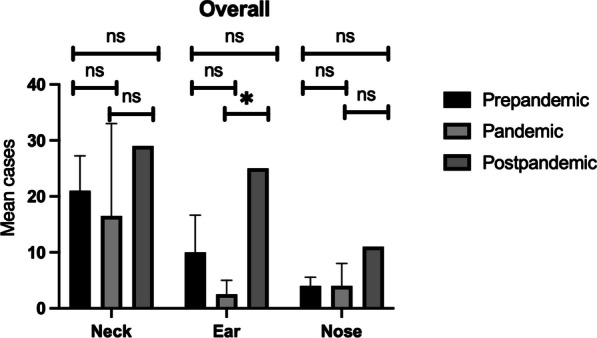
Overall mean number of upper respiratory tract infection complications requiring surgical intervention

Statistical analysis revealed a statistically significant increase (*p *value = 0.03) in ear surgeries between the pandemic and post-pandemic groups (Fig. 1). All other analyses comparing pre-pandemic, pandemic, and post-pandemic groups revealed no statistically significant differences. The most common interventions were as follows: for complicated head and neck infections, tonsillectomy was the most common interventions, followed by cervicotomy and transoral drainage of pharyngolaryngeal abscesses. In case of complicated otologic infections, tympanostomy tube insertion was the most common surgery, followed by mastoidectomy. In case of complicated nasal infections, endoscopic sinus surgery was the most common procedure, followed by combined internal and external sinus surgery.

**Fig. 2:**
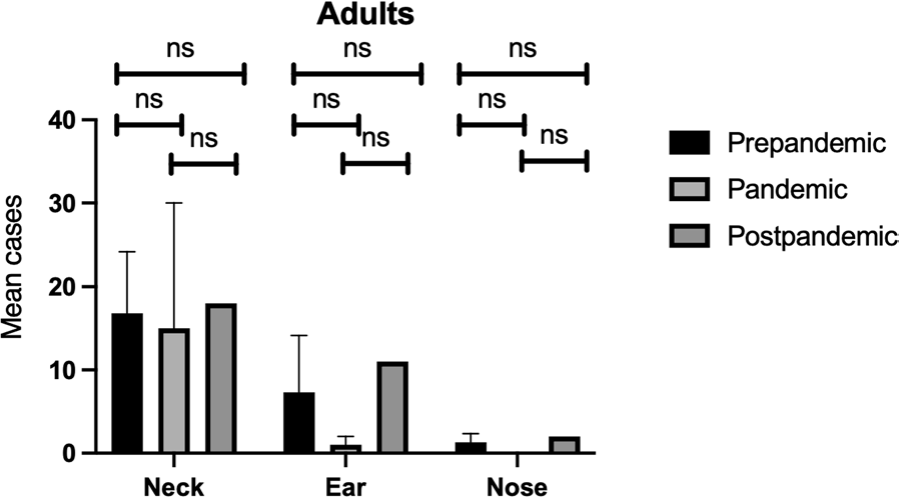
Overall mean number of adults with upper respiratory tract infection complications requiring surgical intervention

**Fig. 3:**
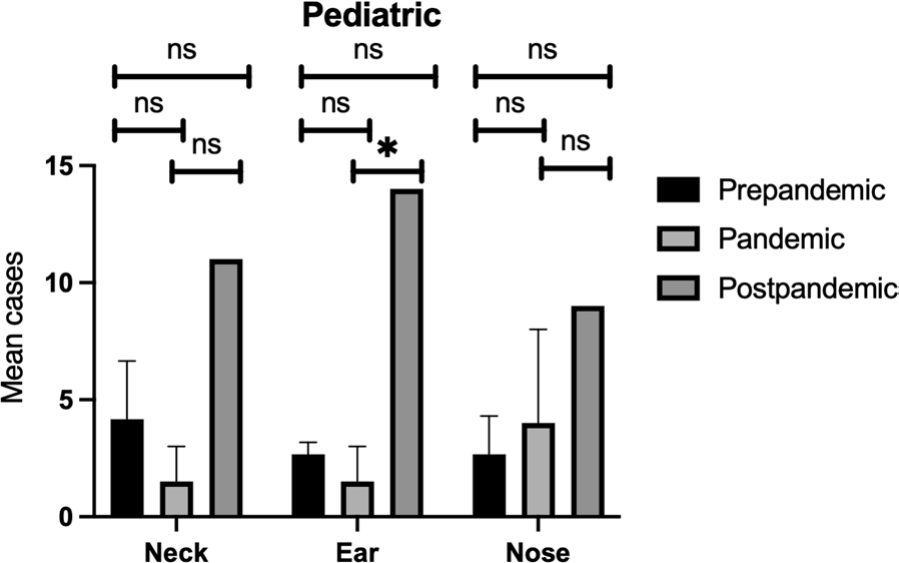
Overall mean number of children with upper respiratory tract infection complications requiring surgical intervention

Further age-related analysis demonstrated no statistically significant differences in adults between the different groups (Fig. 2). However, in the pediatric population, there was a statistically significant increase (*p* value = 0.02) in ear surgery from the pandemic to the post-pandemic group (Fig. 3).

Analysis of follow-up examinations showed no statistically significant differences between the pre-pandemic, pandemic and post-pandemic groups regarding the restitutio ad integrum, subsequent treatments, or consecutive injuries.

**Fig. 4:**
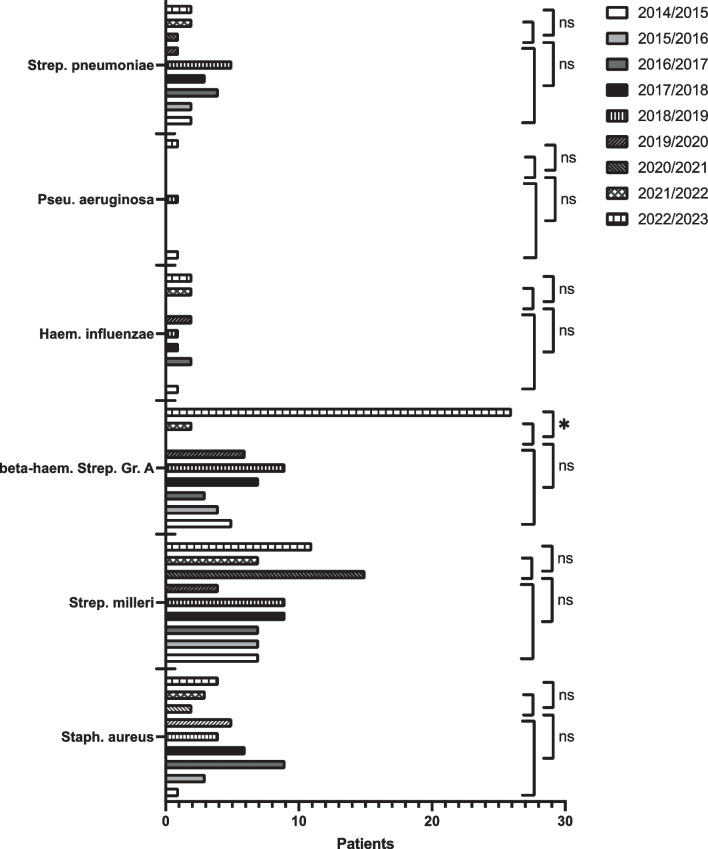
Overall number of typical pathogens of upper respiratory tract infections

Evaluation of the characteristic pathogens for upper respiratory tract infections demonstrated a statistically significant increase (*p *value = 0.02) of beta-hemolytic group A streptococci as pathogens comparing the pandemic to post-pandemic groups (Fig. 4). No statistically significant differences were observed for the other pathogens between the groups.

## Discussion

In this study, we assessed the consequences of pandemic-related measures and their subsequent relaxation on the incidence and characteristics of upper respiratory tract infection-related complications. The main findings were: (1) the statistically significant increase in complicated middle ear infections requiring surgical intervention in the post-pandemic period; (2) this increase was mainly due to a massive increase in the pediatric group; (3) no significant differences in the other procedures or demographics were observed and (4) the prevalence of beta-hemolytic group A streptococcal infections was statistically significantly increased in the post-pandemic group**.**

The significant increase in complicated otologic infections in the post-pandemic period compared to the pandemic period suggests that the relaxation of COVID-19-related restrictions may have contributed to a higher rate of complicated upper respiratory tract infections requiring surgical management. However, no statistically significant differences were observed in complicated nose or neck infections or demographic parameters. Furthermore, the restrictions implemented did not show a statistically significant reduction in the number of complicated upper respiratory tract infections during the pandemic period compared with the pre-pandemic period and pre-pandemic period with post-pandemic period.

The significant increase of complicated ear infections in the pediatric population suggests that children may have been more susceptible to complications of upper respiratory tract infections after the COVID-19 pandemic. Various factors might be relevant, such as a weaker immune system due to lack of exposure and maturation, proliferation of a different spectrum of bacteria-subtype or increase in viral co-infections. There might also be a catch-up effect from previously prevented infections due to the pandemic-related restrictions. However, our analysis of characteristic pathogens associated with upper respiratory tract infections revealed a statistically significant increase only in beta-hemolytic group A streptococcal infections in the post-pandemic period compared with the pandemic period. In the case of a previously mentioned catch-up effect, we would not expect a clear increase of only one bacterial species. Furthermore, the number of other bacterial species did not show any significant differences in the pre-pandemic, pandemic and post-pandemic period. This finding may indicate a shift in the bacterial profile of complicated upper respiratory tract infections following the termination of pandemic-related measures. A similar observation was reported recently, presenting an increase in invasive group A streptococcal infections in young children in the Netherlands in 2022 [11]. Proliferation of a different subtype of bacteria might influence the increased complications of upper respiratory tract infections, although subtyping of *S. pyogenes* revealed that no specific subtype is responsible for the increased incidence [11].

Another possible explanation for the increase of beta-hemolytic group A streptococcal infections might be the lack of exposure of microbial pathogens to the immune system during the pandemic period. In this context, Cohen et al. suggested that the longer the periods of low microbial exposure, the greater the risk for future epidemics, due to an increase in the proportion of susceptible individuals and an induced immunity debt in the population [12]. To reduce this susceptibility to certain pathogens due to pandemic-related measures, regular boosting of the innate immune system with regular vaccination might be able to close such an induced immunity debt [13].

Another contributing factor leading to complications in upper respiratory tract infections might be the increase in viral co-infections. As recently shown with invasive pneumococcal disease, an increase in invasive pneumococcal disease was associated with an increase in respiratory viruses’ disease in children after lifting pandemic-related restrictions [14]. Therefore, it is possible that viral co-infection might also contribute to the increase in beta-hemolytic group A streptococcal infections, although there was no standardized viral pathogen testing in our cohort.

Last but not least healthcare seeking behavior during the pandemic was different and progression of disease in case of chronic rhinosinusitis were reported [3]. At present, however, it is not yet known whether healthcare-seeking behavior in the post-pandemic period is also still altered compared to the pre-pandemic period and whether, as a result, treatment of infections is postponed.

Therefore, it is important to further investigate the factors contributing to the increase in complicated upper respiratory tract infections and investigate possible interventions to mitigate such complications in children to address similar effects in future pandemic-related measures for children.

Nevertheless, our study has several limitations. First, it is a retrospective analysis based on surgical records, which might introduce selection bias and limit the validity of the findings. Second, the study was conducted in a single tertiary referral center, and the results might not be representative of other regions or health care settings. Third, bacterial subtyping and assessment of viral co-infection were not performed.

Future research should aim to address these limitations and further investigate the impact of the pandemic-related measures on complicated upper respiratory tract infections. Further studies involving multiple centers and larger sample sizes including bacterial subtyping and assessment of viral co-infections would provide more robust evidence.

## Conclusions

Our study highlights the potential impact of the COVID-19 pandemic on complications of upper respiratory tract infections requiring surgical intervention. The observed increase in complicated otologic infections in the post-pandemic period, particularly in children, raises concerns about the long-term effects of pandemic-related measures. Understanding these trends will enable healthcare providers to adapt management strategies, allocate resources effectively and implement preventive measures to reduce complications of upper respiratory tract infections in the post-pandemic period.

## Data Availability

Anonymized version of the data is available upon request.
