# Correction: Impact of COVID-19 pandemic: increase in complicated upper respiratory tract infections requiring ENT surgery?

**DOI:** 10.1007/s00405-024-08490-7

**Published:** 2024-01-24

**Authors:** Jonas Galli, Sean C. Sheppard, Marco Caversaccio, Lukas Anschuetz, Sven Beckmann

**Affiliations:** Department of Otorhinolaryngology, Head and Neck Surgery, Inselspital, Bern University Hospital, University of Bern, Freiburgstrasse 18, 3010 Bern, Switzerland

**Correction: European Archives of Oto-Rhino-Laryngology** 10.1007/s00405-023-08349-3

In the original publication of the article, the author’s name Sean C. Sheppard was incorrectly written as Sean S. Sheppard.

The original article was updated.

